# Liver-specific ceramide reduction alleviates steatosis and insulin resistance in alcohol-fed mice[S]

**DOI:** 10.1194/jlr.RA119000446

**Published:** 2020-05-12

**Authors:** Jason Correnti, Chelsea Lin, Jascha Brettschneider, Amy Kuriakose, Sookyoung Jeon, Eleonora Scorletti, Amanke Oranu, Dru McIver-Jenkins, Isabelle Kaneza, Delfin Buyco, Yedidya Saiman, Emma E. Furth, Josepmaria Argemi, Ramon Bataller, William L. Holland, Rotonya M. Carr

**Affiliations:** * Division of Gastroenterology, University of Pennsylvania, Philadelphia, PA; § Department of Pathology and Laboratory Medicine, University of Pennsylvania, Philadelphia, PA; † Division of Gastroenterology, United Health Services, Binghamton, NY; ** Center for Liver Diseases, Pittsburgh Research Center, University of Pittsburgh Medical Center, Pittsburgh, PA; †† Department of Nutrition and Integrative Physiology, University of Utah, Salt Lake City, UT

**Keywords:** alcoholic liver disease, lipid droplets, lipophagy

## Abstract

Alcohol’s impairment of both hepatic lipid metabolism and insulin resistance (IR) are key drivers of alcoholic steatosis, the initial stage of alcoholic liver disease (ALD). Pharmacologic reduction of lipotoxic ceramide prevents alcoholic steatosis and glucose intolerance in mice, but potential off-target effects limit its strategic utility. Here, we employed a hepatic-specific acid ceramidase (ASAH) overexpression model to reduce hepatic ceramides in a Lieber-DeCarli model of experimental alcoholic steatosis. We examined effects of alcohol on hepatic lipid metabolism, body composition, energy homeostasis, and insulin sensitivity as measured by hyperinsulinemic-euglycemic clamp. Our results demonstrate that hepatic ceramide reduction ameliorates the effects of alcohol on hepatic lipid droplet (LD) accumulation by promoting VLDL secretion and lipophagy, the latter of which involves ceramide cross-talk between the lysosomal and LD compartments. We additionally demonstrate that hepatic ceramide reduction prevents alcohol’s inhibition of hepatic insulin signaling. These effects on the liver are associated with a reduction in oxidative stress markers and are relevant to humans, as we observe peri- LD ASAH expression in human ALD. Together, our results suggest a potential role for hepatic ceramide inhibition in preventing ALD.

The rise in cirrhosis-related mortality since 2009 in the United States is due largely to alcoholic liver disease (ALD) with those between the ages of 25 and 34 experiencing the most significant increase. Of these, women are most impacted (1). ALD progresses from alcoholic steatosis to steatohepatitis, fibrosis, and cirrhosis, and this progression is associated with insulin resistance (IR) (2, 3), the key determinant of glucose intolerance in ALD patients (4). Because only a small percentage of patients with alcohol dependence or overuse are able to achieve abstinence (5–7), therapeutic strategies that target alcohol’s hepatic and dysmetabolic effects at the stage of steatosis are needed to prevent the development of advanced disease.

A key feature of alcoholic liver injury is the impairment of both hepatic lipid metabolism and hepatic insulin signaling. Indeed, we have demonstrated that the onset of lipid dysregulation and resultant alcoholic steatosis is temporally related with the onset of IR in alcohol-fed mice (8). We have demonstrated further that these physiologic perturbations require the presence of the major hepatocellular lipid droplet (LD) protein, perilipin 2 (PLIN2) (9), and the accumulation of hepatic ceramides (8–10), bioactive sphingolipids that can impair cell growth, promote apoptosis, and impair insulin signaling (3, 11, 12).

Hepatic ceramides are increased in ALD patients (13), and we and others have demonstrated that long-chain hepatic ceramides are increased in ALD rodent models (8, 9, 13–17). Ceramides are synthesized via three major pathways: *1*) de novo synthesis resulting from the condensation of serine and palmitate in the ER by the pathway’s rate limiting enzyme, serine palmitoyl transferase; *2*) lysosomal salvage due to the reacylation of sphingosine derived from more complex sphingolipids; and *3*) sphingomyelin hydrolysis. We recently established that ceramides are present in the LD fraction and regulate *Plin2* gene transcription through the enzyme ceramide synthase (10), whose activity is required for both ER de novo and lysosomal ceramide synthesis. Lysosomal ceramides can be subsequently deacylated into the ceramide precursor, sphingosine, by ceramidases.

There are currently no suitable experimental rodent models that replicate the progression from alcoholic steatosis to advanced chronic ALD. The Lieber-DeCarli chronic alcohol feeding model is a well-validated model of alcoholic steatosis in the setting of ongoing alcohol consumption but does not model advanced liver disease (18, 19). Using this model, we demonstrated previously that systemic pharmacologic reduction of ceramides prevents alcoholic steatosis and glucose intolerance in mice (10), but off-target effects of this strategy limit its utility. An inducible model of hepatic acid ceramidase (ASAH) overexpression and ceramide deacylation has been developed previously and used to demonstrate improved insulin sensitivity and hepatic steatosis in high-fat diet-fed mice (20). We employed this model of hepatic-specific ASAH overexpression to reduce hepatic ceramides in mice fed alcohol chronically. Here we report that hepatic ceramide reduction via ASAH overexpression ameliorates alcoholic steatosis and improves hepatic insulin sensitivity; and that this enzyme is relevant in human patients with ALD. We additionally establish that there is inter-organelle cross-talk between the lysosomal and LD ceramide pools and that this cross-talk is mediated by lipophagy.

## MATERIALS AND METHODS

### Animal studies

Experiments were performed according to the protocols approved by the Institutional Animal Care and Use Committee of the University of Pennsylvania. All efforts were made to minimize animal discomfort and animals were treated with humane care. Inducible, liver-specific ASAH-transgenic “ASAH+” mice were a generous gift of Drs. William Holland and Phillip Scherer while Dr. Holland was at the University of Texas Southwestern (20). ASAH+ mice have liver-specific inducible expression of *N*-acylsphingosine amidohydrolase 1 (ASAH1), accomplished by three transgenes: Rosa26-loxP-stop-loxP-reverse tetracycline-controlled transactivator (rtTA), Albumin-Cre, and tetracycline response element (TRE)-ASAH1. Doxycycline (Dox) treatment activates rtTA binding to the TRE and drives transcription of the ceramidase ASAH1, specifically in hepatocytes. While all mice in our experiments are homozygous for the Rosa26-loxP-stop-loxP-rtTA and Albumin-Cre transgenes, only the ASAH+ mice have the TRE-ASAH1 transgene, and the ASAH1 transgene is only expressed in the presence of Dox (20). For all experiments, ASAH+ and ASAH− littermates were used.

Weight-matched 8- to 14-week-old female mice were used for the experiments, with weight distributed equally across the genotypes. We chose female mice to replicate the epidemiologic and experimental observations that females are more susceptible to the effects of alcohol than males (1, 21). To acclimate animals to the ethanol (Etoh) diet, Etoh concentration was increased gradually by 2 days of feeding each at 0, 5, 10, and 15% Etoh-derived calories. Mice were then pair-fed a Lieber-DeCarli 82, Shake and Pour liquid diet containing 28% Etoh-derived calories (Bioserv, Flemington, NJ; 36% fat, 13.5% carbohydrate, 1% protein). Dox mice were supplemented with 200 mg/l Dox hyclate (Sigma-Aldrich, St. Louis, MO). Food intake and body weight were measured twice a week.

Blood glucose was measured using the Accu-Chek Nano glucose meter (Roche Diabetes Care, Inc., New York, NY). To assess insulin-stimulated hepatic AKT serine/threonine kinase (Akt) activation, animals were fasted for 6 h (8:00 AM to 2:00 PM), given 2 mU/g Novolin regular insulin (Novo Nordisc, Plainsboro, NJ) intraperitoneally, and livers were isolated by freeze clamping 20 min after injection.

To estimate hepatic VLDL production, a VLDL kinetic experiment was performed. Mice were fasted for 4 h (7:00 AM to 11:00 AM) prior to injection of 1 g/kg poloxamer 407 (Sigma-Aldrich), which blocks the lipolysis of TGs, thereby enabling estimation of the rate of VLDL secretion (22). Tail vein blood was collected at time 0, and 1, 2, and 4 h for TG measurement by colorimetric assay as described (23). ApoB was measured by ELISA (Abcam, Cambridge, MA).

To inhibit lipophagy, the lysosomal inhibitor leupeptin was administered. Leupeptin (40 mg/kg; Sigma-Aldrich) in PBS was given intraperitoneally to mice 2 h before euthanization and tissue harvest. For all experiments, mice were euthanized by CO_2_ inhalation, and tissue was collected for analysis.

### Metabolic monitoring and NMR for body composition

Mice were individually placed in the Oxymax laboratory animal monitoring system (Columbus Instruments, Columbus, OH). Following a 24 h acclimation period, oxygen consumption, carbon dioxide production, and locomotor activity were determined. Respiratory exchange ratio (RER) was calculated by dividing the volume of carbon dioxide produced by the volume of oxygen consumed (VCO_2_/VO_2_). Whole-body fat and lean mass determinations were performed using the EchoMRI-100 system (EchoMRI; Houston, TX).

### Hyperinsulinemic-euglycemic clamps

Clamp studies were performed at the University of Pennsylvania Diabetes Research Center Mouse Phenotyping, Physiology, and Metabolism Core. Indwelling jugular vein catheters were surgically implanted 5 days prior to the clamp study day as described previously (24). Mice were fasted for 5 h prior to initiation of the clamp and acclimated to the plastic restrainers for tail sampling. A [3-3H] glucose infusion was primed (5-μCi) and continuously infused for a 120 min equilibration period (0.05 μCi/min). Baseline measurements were determined in blood samples collected at -10 and 0 min (relative to the start of the clamp) for analysis of glucose, [3-3H]glucose-specific activity, and basal insulin. The clamp was started at *t* = 0 min with a primed-continuous infusion of human insulin (16 mU/kg bolus followed by 2.5 mU/kg/min; Novolin regular insulin), and glucose (D20 mixed with [3-3H]glucose 0.03 μCi/μl) was infused at a variable glucose infusion rate (GIR) to maintain euglycemia. Blood samples were taken at *t* = 80–120 min for the measurement of [3-3H]glucose-specific activity and clamped insulin levels. After the final blood sample, animals were injected with a bolus of pentobarbital, and quadricep muscle and epididymal adipose tissue were collected and frozen in liquid nitrogen and stored in −20°C for subsequent analysis.

The radioactivity of [3-3H]glucose, [^14^C]2DG, and [^14^C]2DG-6-phosphate were determined as described previously (25). The glucose turnover rate [total rate of endogenous glucose production (Ra); milligrams per kilogram per minute) was calculated as the rate of tracer infusion (disintegrations per minute per minute) divided by the corrected plasma glucose specific activity (disintegrations per minute per milligram) per kilogram of body weight of the mouse. Glucose appearance (Ra) and disappearance [rate of peripheral glucose disposal (Rd)] rates were determined using steady-state equations, and endogenous glucose production (Ra) was determined by subtracting the GIR from total Ra. Tissue-specific glucose disposal (Rg; micromoles per 100 grams of tissue per minute) was calculated as described previously (25).

### Lipid analyses

TGs (Stanbio, St. Boerne, TX), cholesterol (Wako, Mountain View, CA), β-hydroxybutyrate (Stanbio), alanine aminotransferase (ALT; Stanbio), and NEFAs (Wako) were measured using enzymatic colorimetric assays. Liver TGs were measured in Etoh:KOH lipid extracts. For liver ceramide analysis, liver was homogenized in RIPA buffer and quantitated using the Pierce™ BCA protein assay kit (Thermo Fisher Scientific, Waltham, MA). Samples were analyzed by mass spectrometry for ceramide content at the metabolomics cores at Stony Brook University School of Medicine and at the Medical University of South Carolina. Whole and fractionated liver samples were normalized to protein and equal serum volumes were measured. Isolation of LDs, lysosomes, and ER for ceramide analysis was performed as described (26).

### Western blotting

Liver lysates for Western blot were generated by homogenization in RIPA buffer and protein content was assessed by BCA assay (Thermo Fisher Scientific). Protein (50 μg) was separated by SDS-PAGE, transferred, and probed with antibodies specific for PLIN2 (1:1,000; ab108323, Abcam), lysosomal-associated membrane protein 1 (LAMP1) (1:1;000; 3243, Cell Signaling Technology, Danvers, MA), microtubule-associated protein light chain 3 (LC3B) (1:1,000; nb100-2220, Novus Biologicals, Centennial, CO), or GAPDH (1:2,000; MAB374, MilliporeSigma, Burlington, MA). All blots were visualized by chemiluminescence using secondaries conjugated to HRP (1:5,000; sc-2004, Santa Cruz Biotechnology, Santa Cruz, CA), except GAPDH, which was visualized by infrared secondary (1:15,000; 926-68070, LiCor, Lincoln, NE) scanned on a LiCor Odyssey (LiCor). Quantitation was done using LiCor software and on scans of radiographs in ImageJ software (National Institutes of Health, https://imagej.nih.gov/ij/).

### Gene expression

RNA was extracted using the PureLink™ RNA mini kit (Life Technologies, Carlsbad, CA). DNase I-treated (Life Technologies) RNA was reverse transcribed using a high-capacity cDNA reverse transcription kit, and mRNA expression was measured by real-time PCR (Applied Biosystems) using TaqMan primers from Life Technologies. ASAH1 mRNA was quantified using SYBR Green (Applied Biosystems) with PCR primers described previously (20). Relative mRNA expression was normalized to GAPDH, 18s rRNA, or 36B4.

### RNaseq

Total RNA quantity and quality were assayed with an Agilent 2100 bioanalyzer instrument using the RNA 6000 Nano kit (Agilent Technologies). Libraries were prepared at Next Generation Sequencing Core at the University of Pennsylvania using TruSeq Stranded mRNA HT Sample Prep Kit (Illumina) as per the standard protocol in the kit’s sample preparation guide. Libraries were assayed for size using a DNA 1000 kit of Agilent 2100 Bioanalyzer (Agilent Technologies) and quantified using the KAPA Library quantification kit for Illumina platforms (KAPA Biosystems). One hundred base pair single-read sequencing of multiplexed samples was performed on an Illumina HiSeq 4000 sequencer. Illumina’s bcl2fastq version 2.20.0.422 software was used to convert bcl to fastq files.

The Molecular Profiling Facility at the University of Pennsylvania performed data analysis. Raw sequence files (fastq) were mapped using salmon (https://combine-lab.github.io/salmon/) against the mouse transcripts described in GENCODE (version M19, built on the mouse genome GRCm38.p6, https://www.gencodegenes.org). Transcript counts were summarized to the gene level using tximport (https://bioconductor.org/packages/release/bioc/html/tximport.html) and normalized and tested for differential expression using DESeq2 (https://bioconductor.org/packages/release/bioc/html/DESeq2.html). Normalized data were visualized with principal components analysis to assess global relationships among the samples using Partek Genomics Suite (Partek, Inc., St. Louis, MO). A false discovery rate-corrected *P*-value was calculated by DESeq2 using the Benjamini-Hochberg method.

### Histology

Institutional review board-exempt status was obtained to use archived human liver tissue. The protocol conforms to the ethics outlined in the 1975 Declaration of Helsinki. Most samples were fixed in 10% neutral buffered formalin for 24 h and then transferred to 70% Etoh until paraffin embedding. Frozen tissue was thawed, fixed overnight in 10% NBF, transferred to 70% Etoh, and then paraffin embedded for sectioning. Paraffin sections were stained with hematoxylin and eosin. Oil Red O staining on liver sections frozen in cryoprotectant media was performed by the Molecular Pathology and Imaging Core at the University of Pennsylvania. Immunohistochemistry on mouse and de-identified human liver samples was performed using a BOND instrument (Leica Microsystems, Mannheim, Germany) with heat-epitope retrieval for 20 min in ER1 solution. Electron microscopy (EM) sample preparation, immunogold labeling, and imaging were performed by the EM Resource Lab at the University of Pennsylvania.

### Patients and liver RNA sequencing

For human RNaseq studies, human liver samples were obtained from the Human Biorepository Core from the National Institutes of Health-funded international InTeam consortium and from Cliniques Universitaires Saint-Luc (Brussels, Belgium), as described previously (27). All participants gave written informed consent and the research protocols were approved by the local Ethics Committees and by the central Institutional Review Board of the University of North Carolina at Chapel Hill.

A total of 90 patients were included. Patients were grouped into seven categories: nonobese, high alcohol intake, early alcoholic steatohepatitis (“Early”, N = 12); nonsevere alcoholic hepatitis (“Nsev AH”, N = 11); severe alcoholic steatohepatitis (“Sev AH”, N = 29); nondiseased (“Normal”, N = 10); NAFLD without alcohol abuse (“NAFLD”, N = 9); chronic hepatitis C virus (HCV) (HCV, N = 10); and compensated HCV cirrhosis (“Comp HCV Cirr”, N = 9). Patients with malignancies were excluded.

RNA purity and quality assessment, library preparation, sequencing, and bioinformatic analyses have been described previously (27). Total RNA libraries were built using TruSeq Stranded Total RNA Ribo-Zero GOLD (Illumina) and sequenced using the Illumina HiSeq2000 platform. Sequencing was paired end (2 × 100 bp) and multiplexed. Limma package (2) was used for cyclic loss normalization followed by log transformation of the counts per million and mean-variance adjustment using the voom function (28).

### Statistics

Statistical analysis was performed using *t*-test or ANOVA with post hoc Newman-Keuls multiple comparison test (GraphPad Prism, La Jolla, CA). *P* < 0.05 was considered significant in all cases.

## RESULTS

### Hepatic ASAH overexpression reduces hepatic ceramides in alcohol-fed mice

**Fig. 1.**
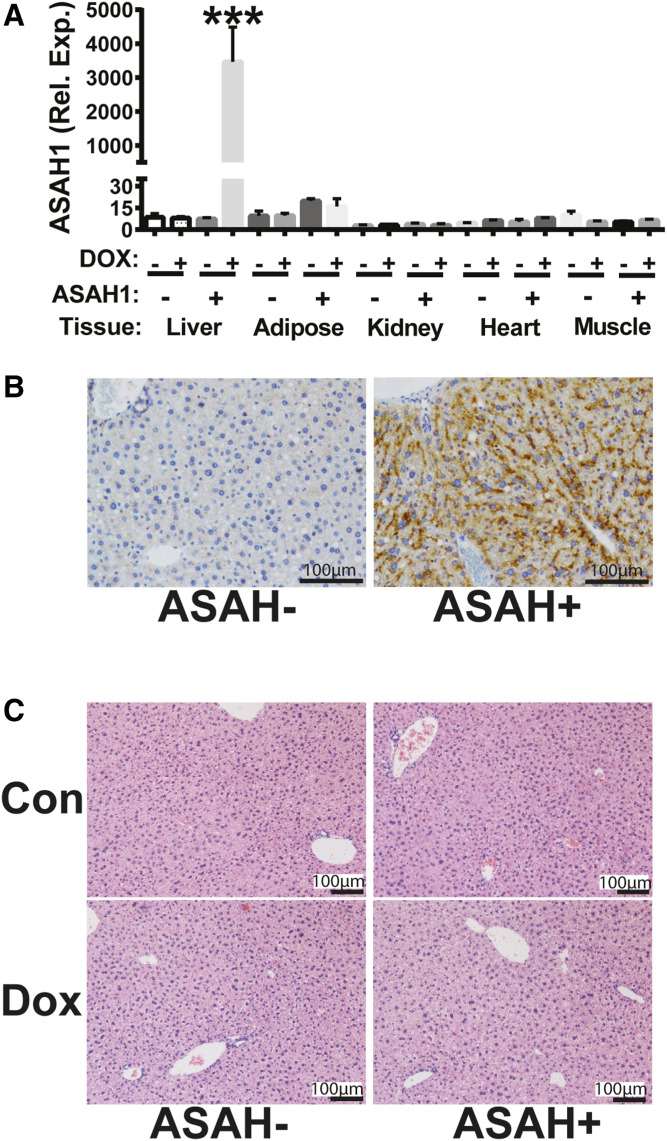
ASAH overexpression is inducible and liver-specific. Female ASAH− and ASAH+ mice were fed a liquid control or Dox-supplemented diet for 7 days. A: ASAH RNA levels from various tissues were assessed by real-time RT-PCR. B: ASAH1 immunohistochemistry in Dox-fed ASAH− and ASAH+ littermates. C: Hematoxylin and eosin stained liver sections. ***P ≤ 0.01.

ASAH is a lysosomal enzyme that hydrolyzes ceramide to sphingosine and a free FA (20). To achieve inducible liver-specific ceramide reduction, ASAH-overexpressing (ASAH+) mice and their control littermates were used for these studies (20). Expression of the ASAH transgene is controlled by rtTA, which is expressed specifically in the liver and is Dox inducible (supplemental Fig. S1). ASAH− mice lack the ASAH transgene. We first confirmed that Dox feeding induced hepatic ASAH mRNA. ASAH− and ASAH+ littermates were ad libitum fed a control liquid diet with Dox for 7 days, and mRNA was measured in various organs. Dox treatment robustly increased ASAH transcript specifically in the livers of ASAH+ mice (**Fig. 1A**). ASAH transgene induction was additionally confirmed by immunohistochemistry (Fig. 1B). Notably, liver histology was similar in ASAH+ and ASAH− control-fed mice (Fig. 1C).

**Fig. 2.**
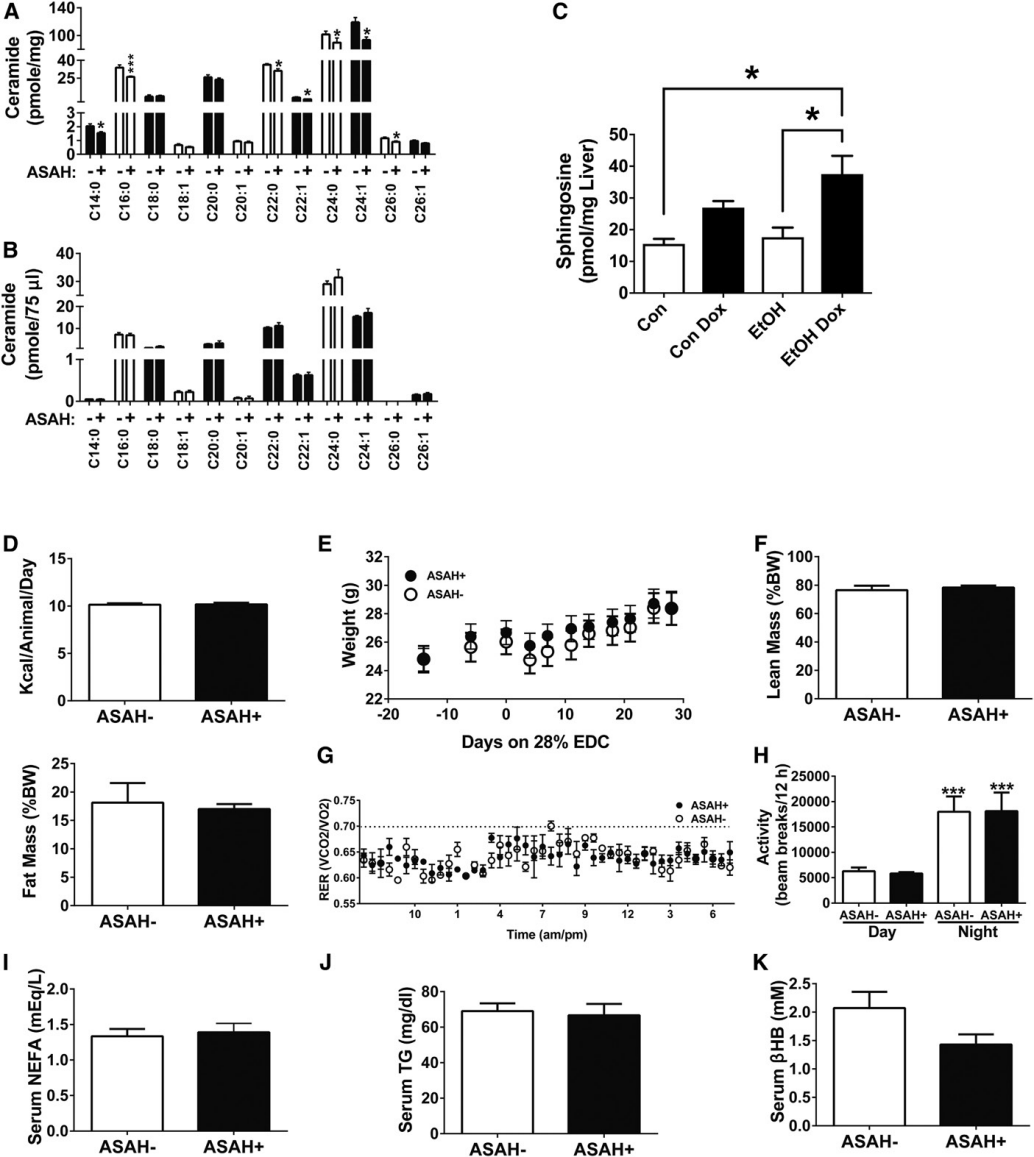
Hepatic ASAH overexpression reduces hepatic ceramides in alcohol-fed mice. Female ASAH− and ASAH+ mice were fed an Etoh-Dox diet for 4 weeks. Ceramide content in the liver (A) and serum (B) was measured by tandem mass spectrometry. C: Hepatic sphingosine content in control (Con), control Dox (Con Dox), alcohol (Etoh), and alcohol Dox (Etoh Dox) mice. Diet consumption (D), weight gain (E), and lean and fat mass (F) measured by NMR in ASAH− and ASAH+ mice. The Comprehensive Lab Animal Monitoring System was used to measure RER (G) and activity (H). Serum NEFA (I), TG (J), and β-hydroxybutyrate (βHB) (K). *P ≤ 0.05; ***P ≤ 001.

As a functional measure of increased ASAH activity, we performed mass spectrometry to quantify liver sphingolipids. Mice were pair-fed a Lieber-DeCarli control or Etoh diet with Etoh accounting for 28% of total caloric intake and supplemented with Dox for 28 days. The Lieber-DeCarli diet is a well-established model of the early stages of alcoholic disease because it induces hepatic steatosis and oxidative stress as a result of chronic alcohol intake. However, this model does not replicate advanced liver injury (19, 29). Relative to Etoh alone, ASAH induction reduced the long chain ceramides C14:0, C16:0, C22:0, C22:1, C24:0, C24:1, and C26:0 (**Fig. 2A**). Serum ceramide levels were similar between groups, confirming that ceramide reductions were liver-specific (Fig. 2B) and liver sphingosine levels were increased, consistent with the known action of ASAH (Fig. 2C).

### Alcohol-fed ASAH− and ASAH+ mice have similar food intake, fuel utilization, and energy expenditure

Similar to our prior observations in wild-type mice (8, 10), chronic alcohol feeding increased liver TG in alcohol-fed ASAH− mice relative to pair-fed controls (supplemental Fig. S2A). Additionally, alcohol-fed ASAH− mice had a 2-fold increase in serum ALT relative to calorie-matched control diet-fed animals (supplemental Fig. S2A, B) and reduced total fat mass (supplemental Fig. S2C). Dox administration did not alter the phenotypic Etoh response in ASAH− mice (supplemental Fig. S2A, B).

For the subsequent experiments, we chose to compare ASAH− and ASAH+ mice fed an Etoh-Dox diet, a comparison that was the least confounding of all comparisons as assessed by RNaseq principal component analysis (data not shown) and that simulates the clinical scenario of patients with ongoing alcohol consumption.

Both ASAH− and ASAH+ mice fed an Etoh-Dox diet consumed approximately 10 kcal/day and had similar weight gain (Fig. 2D, E). NMR imaging demonstrated a similar percentage of fat and lean mass between ASAH− and ASAH+ littermates (Fig. 2F). To assess whether there were any differences in fuel utilization or activity level, we performed comprehensive laboratory animal monitoring. Fat oxidation is the primary fuel source in Etoh-Dox-fed animals, as evidenced by a RER (VCO_2_/VO_2_) (a measure of fuel utilization) of approximately 0.6 in both genotypes during the 24 h monitoring period (Fig. 2G). In addition, there were no differences in the activity pattern of ASAH− and ASAH+ animals. Namely, daytime activity and an ∼2-fold increase in nocturnal activity were observed in both groups (Fig. 2H). These similarities in energy expenditure and fuel utilization between ASAH− and ASAH+ Etoh-Dox-fed mice are supported by the similar serum NEFA, TG, and β-hydroxybutyrate levels (Fig. 2I–K), the constellation of which reflect fat utilization. Together, these data suggest that liver-specific ceramide reduction does not impair overall energy homeostasis in Etoh-Dox-fed mice.

### Liver-specific ceramide reduction reverses alcohol-induced hepatic IR

**Fig. 3.**
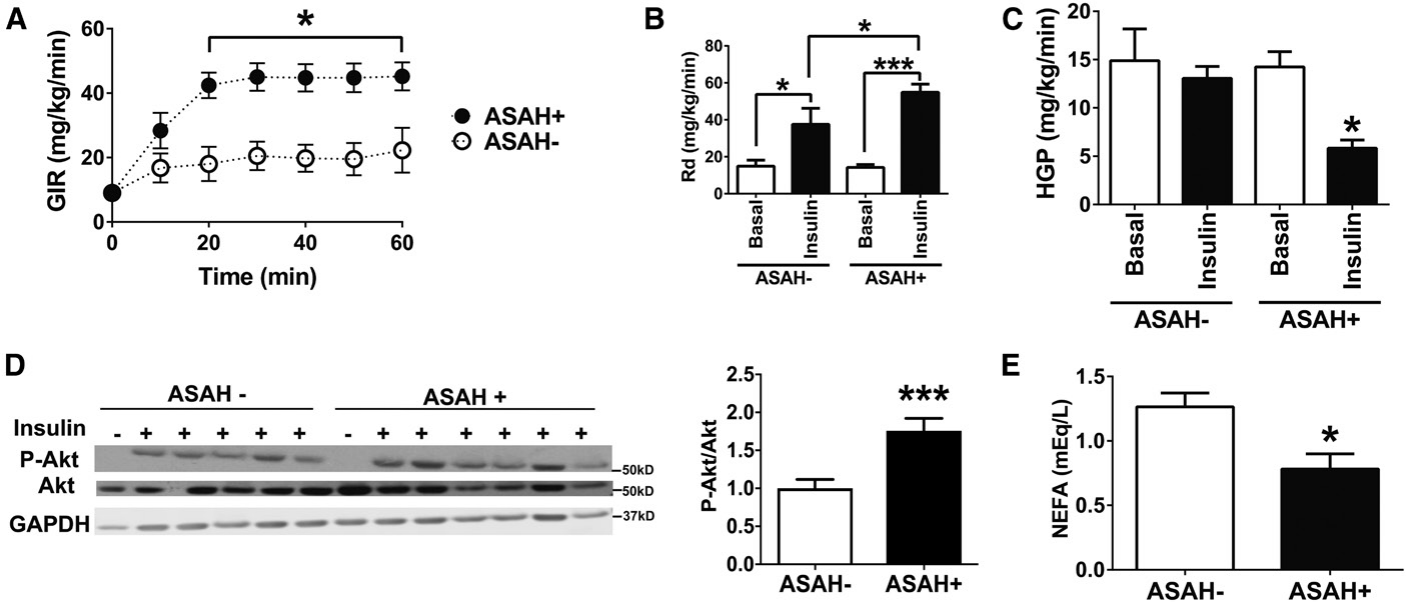
Ceramide reduction reverses alcohol-induced hepatic IR. Female ASAH− and ASAH+ mice were fed an Etoh-Dox diet for 4 weeks and hyperinsulinemic-euglycemic clamps were used to measure GIRs (A), Rd (B), and HGP (C). D: Immunoblots and quantification of Akt and P-Akt from whole liver extracts from mice 20 min after intraperitoneal insulin injection. E: Serum NEFA levels measured during hyperinsulinemic-euglycemic clamp. *P ≤ 0.05; ***P ≤ 0.01.

To assess insulin sensitivity, we performed the gold-standard hyperinsulinemic-euglycemic clamp study. Similar to findings in Etoh-fed wild-type mice (8), clamp GIR, a marker of whole-body insulin sensitivity, was significantly reduced in ASAH− Etoh-Dox-fed mice. GIR was 2-fold higher in ASAH+ mice relative to ASAH− animals (**Fig. 3A**), showing that hepatic ASAH overexpression improves insulin sensitivity in alcohol-fed mice.

The insulin-sensitizing effect of ceramidase overexpression was in part due to improvements in Rd (Fig. 3B). However, the major source of improved insulin sensitivity in ASAH+ Etoh-Dox-fed mice was augmented hepatic insulin sensitivity. Specifically, ASAH− Etoh-Dox-fed mice were refractory to insulin’s inhibitory effects on hepatic glucose production (HGP), while ceramidase-overexpressing mice exhibited a 57% reduction in HGP in response to insulin (Fig. 3C).

To further examine the molecular basis for the improvement in hepatic insulin sensitivity in Etoh-Dox-fed ASAH-overexpressing mice, we quantified hepatic expression of Akt and P-Akt (the major insulin signaling molecules) in response to acute insulin injection. The livers of Etoh-Dox-fed ASAH− and ASAH+ mice were harvested twenty minutes after an intraperitoneal injection of insulin for immunoblot measurement of Akt and P-Akt. Relative to total Akt, ASAH+ mice had a nearly 2-fold increase in P-Akt, demonstrating that ceramide reduction improves hepatic insulin signaling in alcohol-fed mice by an Akt-mediated mechanism (Fig. 3D).

We next measured serum NEFA under clamp conditions, as insulin inhibits adipose lipolysis and subsequent NEFA release, which has been demonstrated to acutely promote HGP (30, 31). Clamp NEFA levels were reduced 38% in ASAH+ mice (Fig. 3E). Together, these data demonstrate that hepatic ceramide reduction improves insulin sensitivity in alcohol-fed mice by increasing both hepatic and adipose insulin signaling.

### Hepatic ceramide reduction improves alcoholic steatosis

**Fig. 4.**
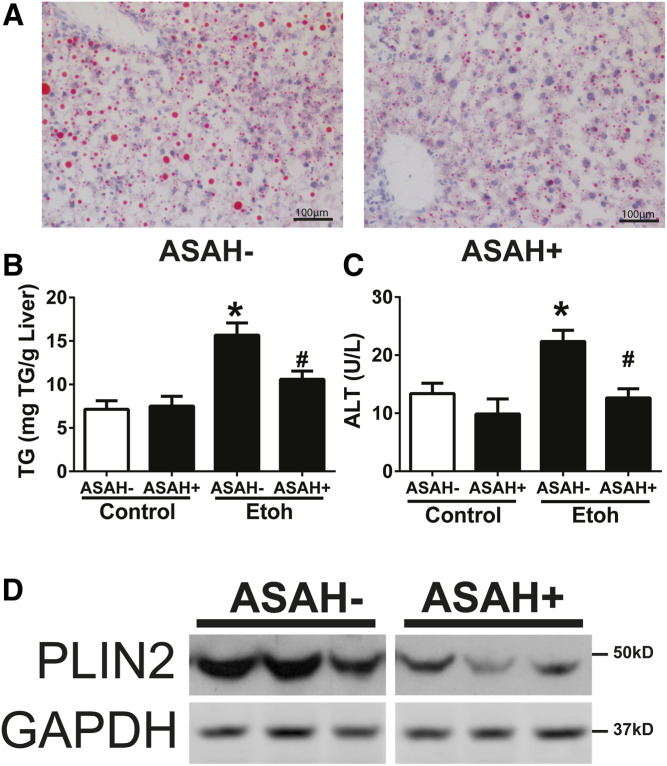
Hepatic ceramide reduction improves alcoholic steatosis. Female ASAH− and ASAH+ mice were fed an Etoh-Dox diet for 4 weeks. A: Oil Red O staining on frozen liver sections. TG levels in whole liver extracts (B), serum ALT (C), and immunoblots (D) from whole liver extracts. *P ≤ 0.05 versus ASAH− Control; #*P* ≤ 0.05 versus ASAH− EtOH.

We demonstrated previously that compared with a control liquid diet, an alcohol liquid diet promotes steatosis, PLIN2 upregulation, and ceramide accumulation in a temporal pattern (8). Here, we examined how hepatic ceramide reduction influences alcohol-induced LD accumulation and PLIN2 upregulation. Etoh-Dox-fed ASAH+ mice had reduced steatosis relative to ASAH− littermates, as evidenced by reduced Oil Red O staining of liver histological sections (**Fig. 4A**) and a 2-fold reduction in liver TG (Fig. 4B). ASAH+ mice also had less hepatocellular injury as assessed by serum ALT (Fig. 4C). Protein expression for PLIN2 (9) was also reduced (Fig. 4D). Together, these data demonstrate that hepatic ceramide reduction reduces LD accumulation and hepatic injury in early ALD.

### Ceramide reduction increases VLDL secretion

**Fig. 5.**
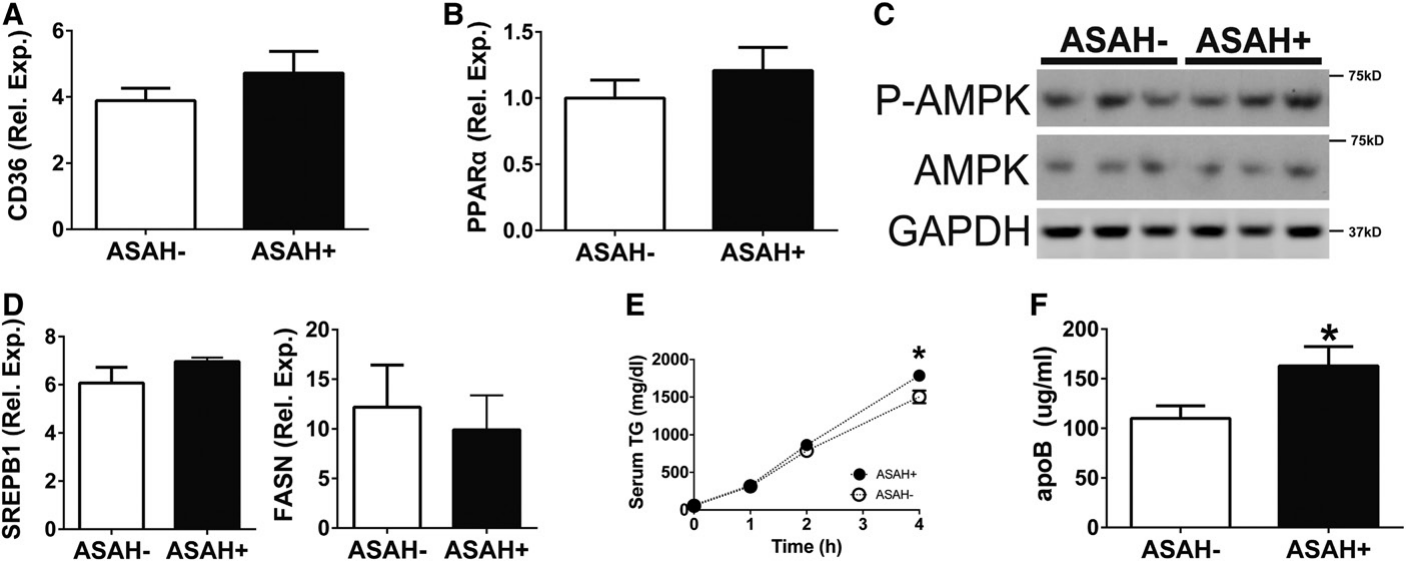
Ceramide reduction increases VLDL secretion. Female ASAH− and ASAH+ mice were fed an Etoh-Dox diet for 4 weeks. RNA extracted from whole liver was assayed by real-time RT-PCR for CD36 (A) and PPARα (B). Immunoblots from whole liver extracts (C) and real-time RT-PCR (D). Serum TG levels during a VLDL kinetic assay (E) and apoB by ELISA (F). *P ≤ 0.05.

To determine the mechanism by which ceramide reduction alleviates steatosis, we analyzed key FA synthetic and metabolic pathways. Ceramide reduction had negligible effects on markers of FA import, oxidation, and synthesis. To examine FA import, we measured RNA levels for the FA transport protein CD36 (**Fig. 5A**) and serum NEFA (Fig. 2I) and found no differences between the ASAH− and ASAH+ mice. For FA oxidation, we observed similar RER (Fig. 2G), serum ketones (Fig. 2K), and hepatic RNA levels of PPARα (Fig. 5B), a transcriptional factor that regulates FA oxidation. Additionally, we saw no differences by Western blot in a key FA oxidative regulator, phosphorylated AMP-activated protein kinase (Fig. 5C). Collectively, these data suggest that FA oxidation was not modulated by hepatic ceramide reduction. To assess FA synthesis, we measured RNA levels of SREBP-1c (a master regulator of de novo lipogenesis) and its target gene, FA synthase, and found no differences (Fig. 5D), suggesting that hepatic ceramide reduction did not alter FA synthesis. Further, RNaseq analysis showed no changes in lipid metabolism genes due to ASAH induction (supplemental Table S1). By contrast, when we measured VLDL TG secretion in vivo, we found an almost 20% increase and a parallel 50% increase in serum levels of the major VLDL protein apoB in ASAH+ mice compared with ASAH− mice (Fig. 3E).. Together, these data demonstrate that hepatic ceramide reduction improves alcoholic steatosis in part through increased hepatic VLDL secretion.

### ASAH induction decreases LD ceramides and increases lipophagy

**Fig. 6.**
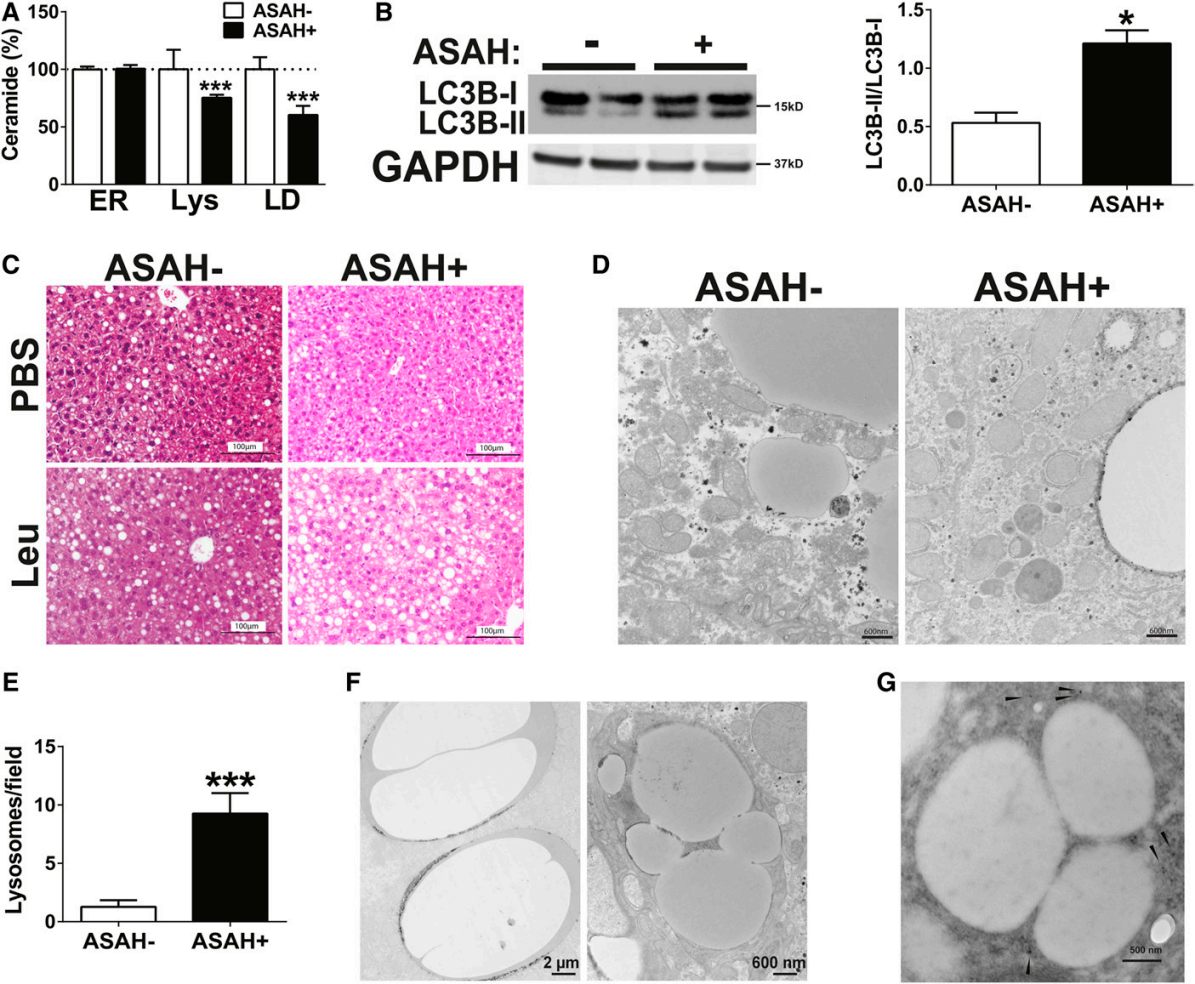
ASAH induction decreases LD ceramides and increases lipophagy. Female ASAH− and ASAH+ mice were fed an Etoh-Dox diet for 4 weeks. A: ER, lysosomes (Lys), and LDs were isolated from liver and assayed by mass spectrometry for ceramide content. B: Immunoblot images from whole liver lysates. C: Liver histologic sections following treatment with intraperitoneal leupeptin or PBS. D: Liver ultrastructure was visualized by EM (scale bar = 600 nm). E: Lysosomes per field was quantitated in EM images. F: Liver ultrastructure by EM. G: Immunogold staining (arrows) for LAMP1 in liver EM section. *P ≤ 0.05; ***P ≤ 0.01.

Ceramides are present in several cellular organelles and membranes; however, little is known about how pharmacologic or genetic manipulation of hepatic ceramides differentially alters ceramide content in subcellular compartments. To address this, we measured ceramides in purified lysosomes, ERs, and LDs (supplemental Fig. S3) from Etoh-Dox-fed mice using a protocol recently described by our laboratory that demonstrates our ability to isolate these organelles without contamination (26). As anticipated, induction of the lysosomal protein ASAH1 reduced lysosomal ceramide levels by approximately 25% (**Fig. 6A**). Notably, there was no apparent effect on de novo synthesized ceramides as the ER ceramide pool was unaffected in ASAH+ mice. Unexpectedly, we observed the greatest reduction in ceramides in the LD fraction, where ceramide concentration was reduced by 40% (Fig. 6A). The effects on LD ceramides were not due to mislocalization of ASAH1 to LD membranes, as confirmed by Western blot of isolated LDs (supplemental Fig. S3), thus implicating an alternative process by which lysosomal ceramide enzymes influence LD ceramide content.

Lipophagy is an autophagic process by which lysosomes degrade LDs in autophagosome structures (32–34). As lysosomes and LDs interact intimately during this process (32), we hypothesized that the observed lysosomal-LD ceramide cross-talk was due to increased lipophagy. To establish whether lipophagy contributed to our observations, we measured LC3 levels in ASAH+ liver lysates. We observed increased LC3-II levels in ASAH+ mice compared with littermate controls (Fig. 6B). We next inhibited lipophagy with leupeptin and observed that we prevented the anti-steatotic effect of ASAH overexpression with lipophagy inhibition (Fig. 6C). To further investigate whether lipophagy contributed to the improvement in steatosis upon ASAH induction, we examined liver ultrastructure by EM in Etoh-Dox-fed ASAH− and ASAH+ mice. Indeed, in ASAH+ mice but not ASAH− mice we found both a significantly greater number of lysosomes (Fig. 6D, E) and numerous LDs enveloped by double-membrane structures that appeared to be autophagolysosomes in ASAH+ mice (Fig. 6F). Immunogold labeling of the EM sections demonstrated that these double-membrane bound vesicles were positive for the lysosomal marker LAMP1 (Fig. 6G), thus confirming that these structures were LD-containing autophagolysosomes. Together, these data demonstrate that there is ceramide cross-talk between the lysosomal and LD cellular compartments, and that hepatic ceramide reduction via lysosomal ASAH1 induction ameliorates alcoholic steatosis in part by increasing lipophagy.

### ASAH1 is induced in human ALD

**Fig. 7.**
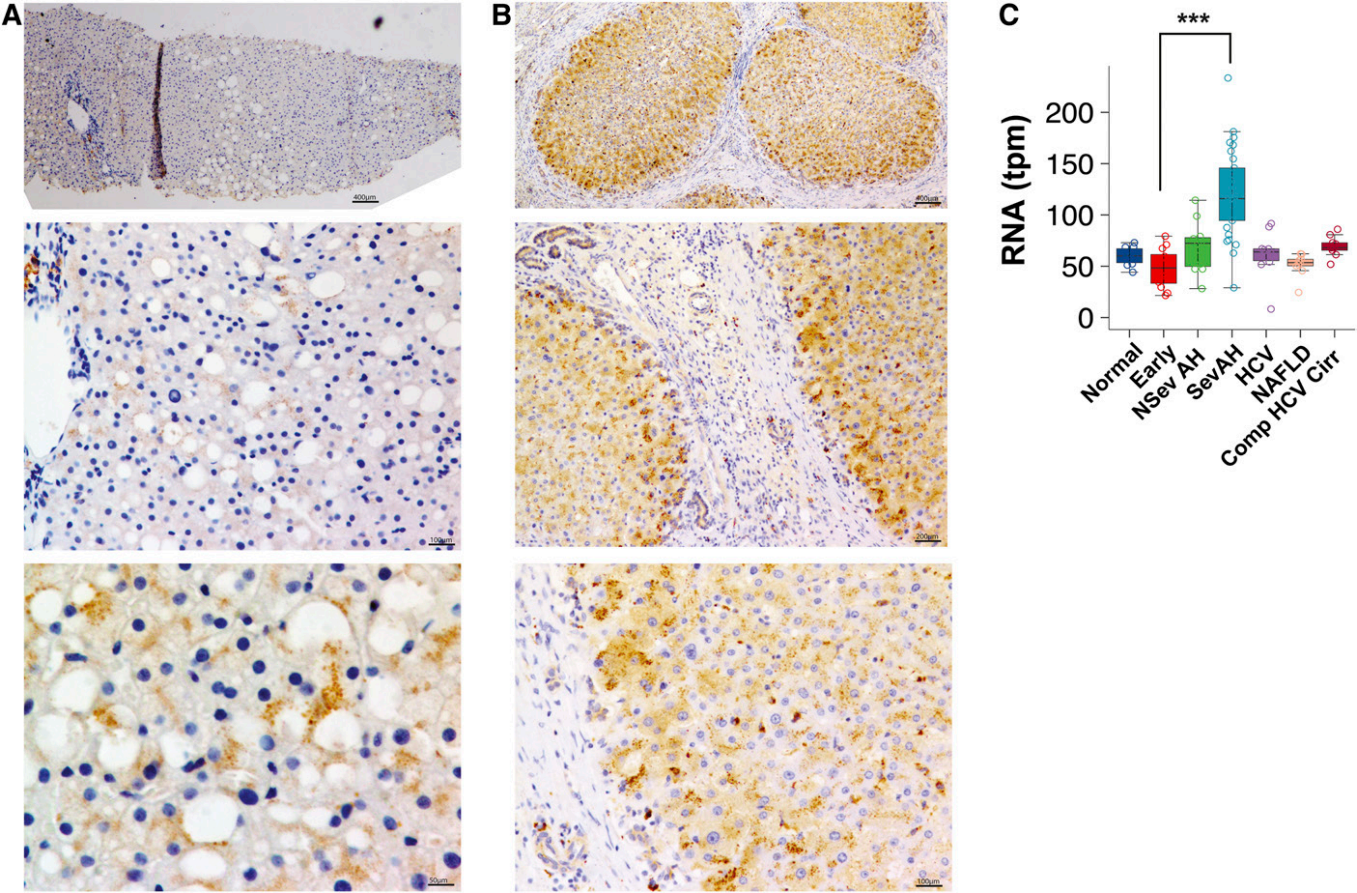
ASAH1 is induced in human ALD. ASAH1 immunostaining in liver biopsies from human alcoholic steatosis (A) and cirrhosis (B). C: Hepatic ASAH1 gene expression in human subjects with no chronic liver disease (Normal), early acute alcoholic hepatitis (Early), nonsevere acute alcoholic hepatitis (NSev AH), severe acute alcoholic hepatitis (Sev AH), chronic HCV, NAFLD, and compensated HCV cirrhosis (Comp HCV Cirr). tpm = transcripts per million. ***P < 0.0007, Sev AH versus Early.

To establish the relevance of ASAH1 induction in human alcoholic steatosis, we performed immunostaining for ASAH1 in human alcoholic liver tissue. We observed that ASAH1 is upregulated in areas surrounding the LD in human liver samples (**Fig. 7A**). ASAH1 expression is also increased in human alcoholic cirrhotic livers, most notably in the hepatocytes that border the fibrous fibroblast-rich regions (Fig. 7B). In addition, in a cohort of patients with and without various etiologies of chronic liver disease (27), patients with severe acute alcoholic hepatitis [a distinct clinical syndrome of ALD that results in high short-term mortality (35)] have the highest levels of hepatic ASAH1 mRNA expression (Fig. 7C). These observations position ASAH1 as not only a regulator of LD biology in early stage disease but also potentially as a modulator of chronic fibro-inflammatory pathways that characterize advanced alcohol-induced liver injury.

**Fig. 8.**
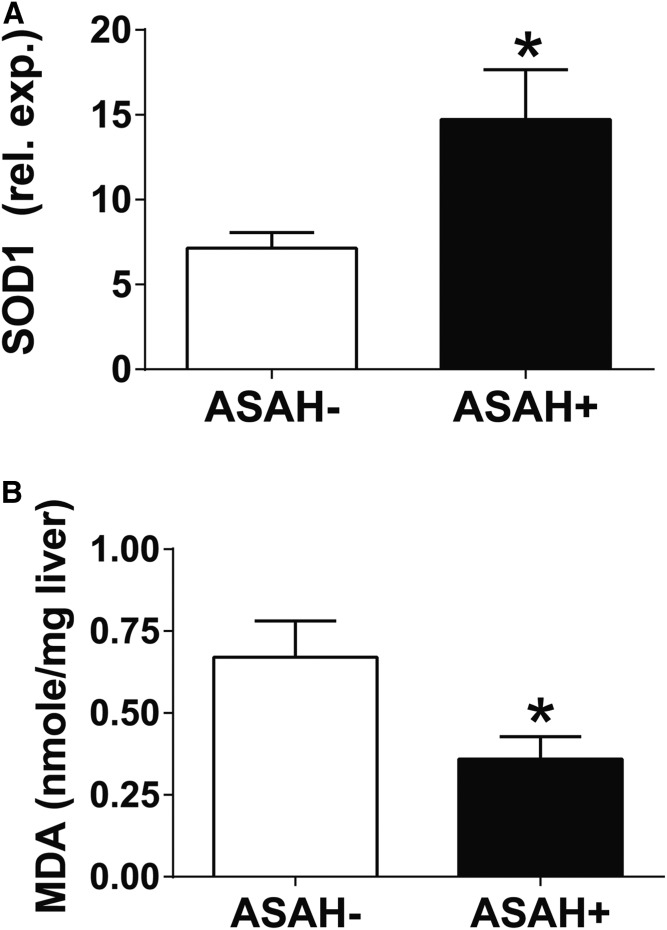
ASAH induction reduces lipotoxicity. Following 4 weeks of Etoh-Dox diet feeding, RNA extracted from whole liver was assayed by real-time RT-PCR for SOD1 (A) and MDA was measured biochemically in whole liver extracts (B). *P ≤ 0.05.

To examine this hypothesis, we performed RNaseq analysis on ASAH− and ASAH+ mice fed an Etoh-Dox diet. This analysis demonstrated differences in only 12 genes between the two groups (supplemental Table S1), including genes involved in oxidative stress [super-oxide dismutase 1 (SOD1)] and ER stress [Derlin 3 (Derl3)]. While the role of Derl3 has not been reported previously in ALD, alcohol-fed mice that lack SOD1 have increased oxidative stress, mitochondrial damage, and liver injury (36). We confirmed the upregulation of SOD1 by RT-PCR (**Fig. 8A**). We, additionally, performed a biochemical assay to determine whether ASAH overexpression improves oxidative stress by quantifying the oxidative stress lipid peroxidation product, malondialdehyde (MDA). Consistent with the RNaseq and RT-PCR results, we observed an approximately 50% reduction in MDA levels in Etoh-Dox-fed ASAH+ mice compared with ASAH− mice (Fig. 8B). These data demonstrate that ASAH1 induction promotes an oxidative stress reduction program and implicates ASAH1 as an important mediator of the liver’s stress response to alcohol overconsumption.

## DISCUSSION

The rising incidence of ALD (1) mandates the incorporation of new mechanistic insights into the paradigm of how we care for patients who suffer from alcohol overconsumption. ALD is a complex metabolic disease and is defined in large part by alcohol’s direct and indirect effects on hepatic lipid metabolism and its systemic effects on glucose tolerance that begin during the stage of hepatic steatosis (3, 37). These perturbations relate ultimately to alcohol’s promotion of cellular stress and the subsequent progression of liver disease from the earliest stage of hepatic steatosis to the more advanced stages of steatohepatitis, fibrosis, and cirrhosis.

In our study, we employed a genetic model of hepatic-specific ceramide reduction (38) that ameliorates the steatotic, insulin desensitizing, and oxidative stress-inducing effects of chronic alcohol intake. We demonstrate that overexpression of the lysosomal enzyme, ASAH, not only reduces hepatic ceramides, as anticipated, but also normalizes hepatic and adipose tissue insulin sensitivity and improves hepatic steatosis despite ongoing alcohol consumption, a clinical scenario faced by the majority of patients who have ALD.

Hepatic steatosis was alleviated by increased VLDL export and lipophagy. Lipophagy is an LD autophagic process negatively regulated by CD36 FA uptake (39) and involves the formation of autophagolysosomes that engulf LDs (32, 40, 41). PLIN2 and ceramides are both regulated by lipophagy (42, 43), and chronic alcohol consumption impairs autophagy in part through inhibition of lysosomal biogenesis and function (10, 44, 45). In our study, increased lipophagy upon ASAH1 induction was demonstrated by a significantly higher lysosomal and autophagolysosomal content in ASAH+ mice compared with ASAH− mice, and the reversal of the anti-steatotic effect with leupeptin. We suspect that the increased lipophagy is linked mechanistically with increased VLDL secretion, as autophagy makes available the lipids needed for apoB stability (46–49).

Enhanced lipophagy as a mechanism of steatosis attenuation upon induction of ASAH1 may relate uniquely to ALD. While reduced FA uptake and suppression of protein kinase C isoform ζ (PKCζ) are the primary mechanisms by which ceramidase overexpression improves steatosis in a nonalcoholic fatty liver model (20), PKCζ is decreased by alcohol (50) and would be an unlikely pathway for further mediation of steatosis. Additionally, based on our prior work showing that reductions in LD ceramides correlate with reduced alcoholic steatosis and IR in PLIN2-KO mice (9), we suspect that it is specifically the reduction of LD ceramides that drives the improvement in hepatic insulin sensitivity in ASAH+ mice.

Our findings are associated with decreased oxidative and lipotoxic stress. Upon Dox induction, alcohol-fed ASAH+ mice have higher mRNA expression of SOD1 and Derl3. While the specific role of Derl3 requires further investigation in the context of ALD (45, 51), the higher SOD1 expression is supported by lower tissue MDA levels [an established marker of lipotoxicity (45)] observed in this study, and higher oxidative stress and liver injury in alcohol-fed SOD1-KO mice demonstrated in a previously published study (36).

Finally, our results have implications for human ALD. In humans with alcoholic steatosis, we observed punctate ASAH1 staining in close apposition to LDs, suggesting lysosome recruitment to the LD. ASAH1 is also upregulated in alcoholic cirrhotic and acute alcoholic hepatitis patients, perhaps as an attempt to reduce oxidative stress in ALD.

In summary, we demonstrate that lysosomal ASAH1 overexpression improves alcohol-induced steatosis, lipid dysregulation, and oxidative stress via hepatic-specific ceramide reduction and that overexpression of this enzyme is relevant in human ALD. We also outline a novel lysosomal-LD ceramide inter-organelle interaction mediated by lipophagy. This strategy of lysosomal ceramide reduction addresses both the metabolic and hepatic-specific effects of alcohol overconsumption and is an example of how molecular approaches can be included in the care of patients with ALD who have ongoing alcohol intake. Future studies will establish the effects of this strategy in more advanced models of ALD, an approach that will ultimately require investigation of positive regulators of ASAH1.

### Data availability

RNaseq datasets from ASAH mice analyzed in the current study are available at GEO, accession #GSE139015. Human RNaseq datasets for ASAH1 have been deposited in the Database of Genotypes and Phenotypes (dbGAP) of the National Center for Biotechnology Information (United States National Library of Medicine, Bethesda, MD) under accession number phs001807.v1.p1. The data supporting this study are available in the article and and the supplementary information, and are available from the corresponding author upon reasonable request.

## Supplementary Material

Supplemental Data
