# Supplementary material for: Liver-specific ceramide reduction alleviates steatosis and insulin resistance in alcohol-fed mice
Source: J Lipid Res. 2020 May 12;61(7):983–94. doi: 10.1194/jlr.RA119000446 (PMC7328039; doi:10.1194/jlr.RA119000446)
Supplement: Supplemental Data [file supp_61_7_983__index.html]

Liver-specific ceramide reduction alleviates steatosis and insulin resistance in alcohol-fed mice — Ceramide reduction improves experimental ALD — Liver-specific ceramide reduction alleviates steatosis and insulin resistance in alcohol-fed mice — Ceramide reduction improves experimental ALD — Liver-specific ceramide reduction alleviates steatosis and insulin resistance in alcohol-fed mice — Supplemental Data 

# Liver-specific ceramide reduction alleviates steatosis and insulin resistance in alcohol-fed mice

## Supplemental Data

- Supporting Information figures and legends
