# Supplementary material for: Liver-specific ceramide reduction alleviates steatosis and insulin resistance in alcohol-fed mice
Source: J Lipid Res. 2020 May 12;61(7):983–94. doi: 10.1194/jlr.RA119000446 (PMC7328039; doi:10.1194/jlr.RA119000446)
Supplement: Supplemental Data [file supp_RA119000446_156273_1_supp_527349_q311zg.pdf]

SUPPLEMENTAL INFORMATION:

Title: Liver-specific ceramide reduction alleviates steatosis and insulin resistance in alcohol-fed mice

Authors:
Jason Correnti, Chelsea Lin, Jascha Brettschneider, Amy Kuriakose, Sookyoung Jeon, Eleonora Scorletti, Amanke Oranu, Dru McIver-Jenkins, Isabelle Kaneza, Delfin Buyco, Yedidya Saiman, Emma E. Furth, Josepmaria Argemi, Ramon Bataller, William L. Holland, Rotonya M. Carr

Jason Correnti, PhD (jacor@pennmedicine.upenn.edu, University of Pennsylvania, Division of Gastroenterology)

Chelsea Lin (chellin@sas.upenn.edu, University of Pennsylvania, Division of Gastroenterology)

Jascha Brettschneider (Jascha@pennmedicine.upenn.edu, University of Pennsylvania, Division of Gastroenterology)

Amy Kuriakose (amykuriakose@gmail.com, University of Pennsylvania, Division of Gastroenterology)

Sookyoung Jeon (Sookyoung.Jeon@pennmedicine.upenn.edu, University of Pennsylvania, Division of Gastroenterology)

Eleonora Scorletti (Eleonora.Scorletti@pennmedicine.upenn.edu, University of Pennsylvania, Division of Gastroenterology)

Amanke Oranu, MD (Oranuamanke@gmail.com, United Health Services, Division of Gastroenterology)

Dru McIver-Jenkins (dhmciverjenkins@gmail.com, University of Pennsylvania, Division of Gastroenterology)

Isabelle Kaneza (kaneza.belle@gmail.com, University of Pennsylvania, Division of Gastroenterology)

Delfin Buyco (Delfin.Buyco@pennmedicine.upenn.edu, University of Pennsylvania, Division of Gastroenterology)

Yedidya Saiman (Yedidya.Saiman@pennmedicine.upenn.edu, University of Pennsylvania, Division of Gastroenterology)

Emma E. Furth, MD (EEF@pennmedicine.upenn.edu, University of Pennsylvania, Department of Pathology and Laboratory Medicine)

Josepmaria Argemi, (j.argemi@pitt.edu, Center for Liver Diseases, Pittsburgh Research Center, University of Pittsburgh Medical Center)

Ramon Bataller, MD, PhD (bataller@pitt.edu, Center for Liver Diseases, Pittsburgh Research Center, University of Pittsburgh Medical Center)

William Holland, PhD (will.holland@hsc.utah.edu, University of Utah, Department of Nutrition & Integrative Physiology)

Rotonya M. Carr, MD (Rotonya.Carr@uphs.upenn.edu, University of Pennsylvania, Division of Gastroenterology)

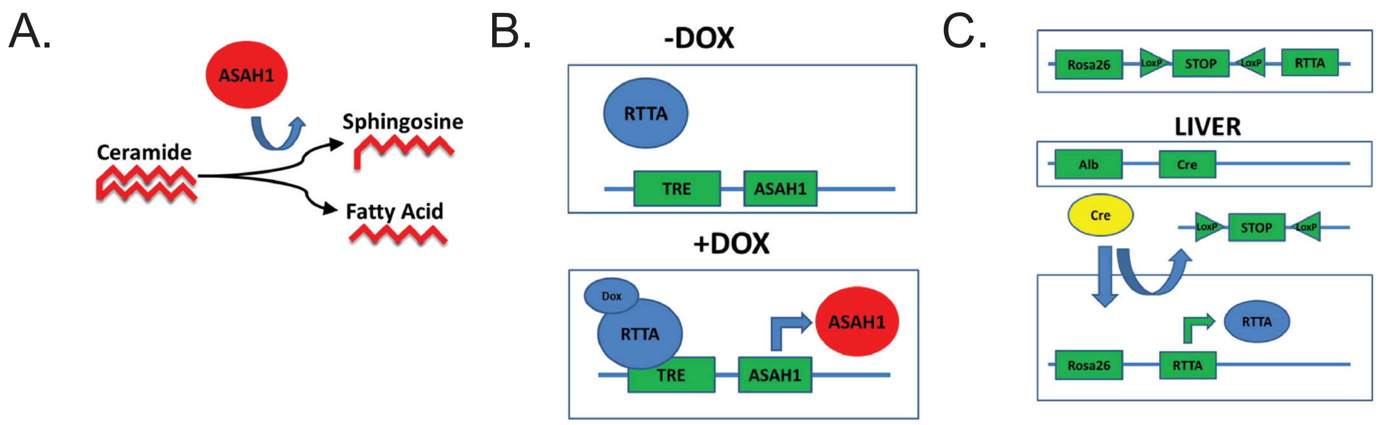

Supplemental Figure S1: Schematic for genetically engineering inducible, liver-specific ceramide reduction in ASAH mice.

(A) The ceramidase ASAH1 reduces ceramide levels by hydrolysis to sphingosine and free fatty acid. (B) ASAH1 transcription is initiated by dox-dependent activation of reverse tetracycline-controlled transactivator (RTTA). (C) RTTA expression is repressed by a loxP-flanked stop codon. Liver-specific expression of Cre recombinase excises the stop codon, allowing RTTA expression exclusively in the liver.

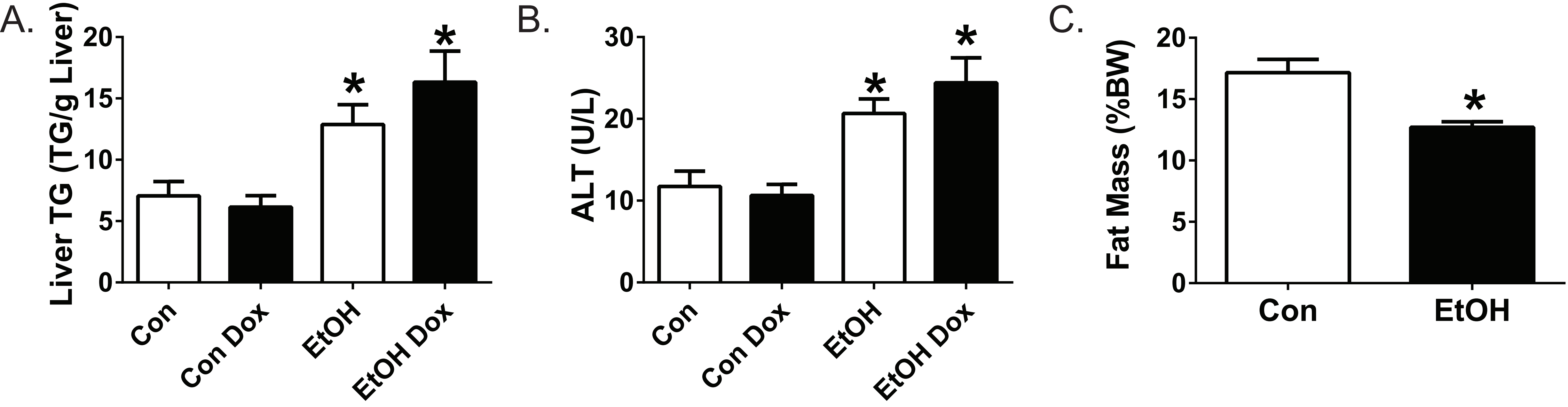

Supplemental Figure S2: Ethanol consumption increases hepatic steatosis in ASAH- mice

Female ASAH- mice were pair-fed a control or ethanol diet with and without dox for 4 weeks. (A) Triglyceride levels in whole liver extracts, (B) serum ALT, and (C) fat mass measured by NMR. *$p \leq 0.05$

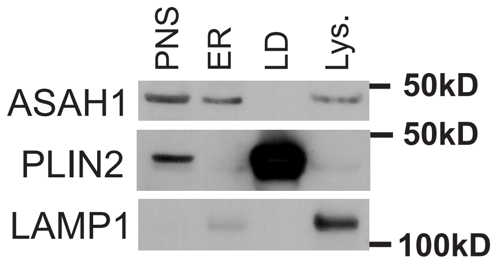

Supplemental Figure S3: ASAH1 overexpression and its subcellular localization. Female ASAH+ mice were fed an Etoh-dox diet for 4 weeks. Whole liver lysate (Post-nuclear supernatant, PNS), endoplasmic reticulum (ER), lysosomes (lys), and lipid droplets (LD) were isolated from liver and assayed by immunoblotting.

Gene symbol	Ensembl Stable ID	Gene description (Source:MGI Symbol)	Gene type	NCBI gene ID	Human gene name	log2 Fold Change	Fold Change	p value	p adj
Asah1	ENSMUSG00000031591	N-acylsphingosine amidohydrolase 1	protein_coding	11886	ASAH1	8.390	335.440	0	0
Sod1	ENSMUSG00000022982	superoxide dismutase 1, soluble	protein_coding	20655	SOD1	0.688	1.611	2.69E-10	2.50E-06
Sel1l	ENSMUSG00000020964	sel-1 suppressor of lin-12-like (C. elegans)	protein_coding	20338	SEL1L	0.460	1.376	1.98E-08	0.0001
Gm45837	ENSMUSG00000030653	predicted gene 45837	protein_coding	207728	PDE2A	-24.158	0.000	1.82E-07	0.0008
Gm4890	ENSMUSG00000097174	predicted gene 4890	lncRNA			2.966	7.815	4.91E-07	0.0018
Gpr12	ENSMUSG00000041468	G-protein coupled receptor 12	protein_coding	14738	GPR12	-2.425	0.186	1.29E-06	0.0040
Col4a5	ENSMUSG00000031274	collagen, type IV, alpha 5	protein_coding	12830	COL4A5	-0.992	0.503	3.35E-06	0.0089
9930021J03Rik	ENSMUSG00000046138	RIKEN cDNA 9930021J03 gene	protein_coding	240613	KIAA2026	0.607	1.523	4.66E-06	0.0108
Derl3	ENSMUSG00000009092	Der1-like domain family, member 3	protein_coding	70377	DERL3	4.036	16.401	6.79E-06	0.0140
Gm10925	ENSMUSG00000100862	predicted gene 10925	pseudogene			2.050	4.140	7.83E-06	0.0145
Slc25a53	ENSMUSG00000044348	solute carrier family 25, member 53	protein_coding	67062	SLC25A53	-2.205	0.217	6.40E-05	0.0991
Zfp991	ENSMUSG00000067916	zinc finger protein 991	protein_coding	666532	ZNF34	-2.052	0.241	6.27E-05	0.0991

Supplemental Table S1: RNAseq analysis reveals ceramide reduction increases SOD1 expression.

Female ASAH- and ASAH+ mice were fed an Etoh-dox diet for 4 weeks. RNA extracted from whole liver was assayed by RNAseq. P-adj is False Discovery Rate corrected p-value, calculated by DESeq2 using the Benjamini Hochberg method. Genes listed have p-adj <0.1.
